# Translating traditional herbal formulas into modern drugs: a network-based analysis of Xiaoyao decoction

**DOI:** 10.1186/s13020-020-00302-4

**Published:** 2020-03-16

**Authors:** Daiyan Zhang, Yun Zhang, Yan Gao, Xingyun Chai, Rongbiao Pi, Ging Chan, Yuanjia Hu

**Affiliations:** 1 State Key Laboratory of Quality Research in Chinese Medicine, Institute of Chinese Medical Sciences, University of Macau, Macau, China; 2 Modern Research Center for Traditional Chinese Medicine, School of Chinese Materia Medica, Beijing University of Chinese Medicine, Beijing, 100029 China; 3 School of Pharmaceutical Sciences, Sun Yat-Sen University, Guangzhou, 510006 China

**Keywords:** Network pharmacology, Traditional Chinese medicine, Xiaoyao decoction

## Abstract

**Background:**

Traditional Chinese medicine (TCM) encompasses numerous herbal formulas which play critical therapeutic roles through “multi-components, multi-targets and multi-pathways” mechanisms. Exploring the interaction among these mechanisms can certainly help to depict the core therapeutic function of herbal formulas. Xiaoyao decoction (XYD) is one of the most well-known traditional Chinese medicine formulas which has been widely applied to treat various diseases. In this study, taking XYD as an example, we proposed a network pharmacology-based method to identify the main therapeutic targets of this herbal concoctions.

**Methods:**

Chemical data of XYD were retrieved from the Traditional Chinese Medicine Systems Pharmacology Database (TCMSP), Traditional Chinese Medicines Integrated Database (TCMID) and Compound Reference Database (CRD) and screened oral bioavailability attributes from SwissADME using Veber’s filter. Targets of sample chemicals were identified using the online tool similarity ensemble approach (SEA), and pathways were enriched using STRING database. On the basis of targets–pathways interactions from the enrichment, a “targets–pathways–targets” (TPT) network was constructed. In the TPT network, the importance of each target was calculated by the declining value of network efficiency, which represents the influential strength of a specific set-off target on the whole network. Network-based predictive results were statistically validated with existing experimental evidence.

**Results:**

The TPT network was comprised of 279 nodes and 6549 edges. The declining value of network efficiency of the sample targets was significantly correlated with their involvement frequency in existing studies of XYD using Spearman’s test (*p* < 0.001). The top 10% of candidate targets, such as AKT1, PIK3R1, NFKB1 and RELA, etc., were chosen as XYD’s main therapeutic targets, which further show pharmacological functions synergistically through 11 main pathways. These pathways are responsible for endocrine, nutritional or metabolic diseases, neoplasms and diseases of the nervous system, etc.

**Conclusions:**

The network pharmacology-based approach in the present study shows promising potential for identifying the main therapeutic targets from TCM formulas. This study provides valuable information for TCM researchers and clinicians for better understanding the main therapeutic targets and therapeutic roles of herbal decoctions in clinical settings.

## Background

Traditional Chinese medicine (TCM), as a unique and complete medical system, was recorded in historical medical documents over 2000 years ago [1]. TCM has been widely used, especially in the form of herbal formulas, which are various combinations of multiple natural herbs [2]. TCM usually plays a therapeutic role on diverse diseases through “multi-components, multi-targets and multi-pathways” [3, 4]. However, it is difficult to decode the molecular evidence of TCM compounds to improve their affinity, specificity, pharmacokinetics and stability [5]. This research gap has restricted the international generalisation and development of TCM [6]. Hence, molecular evidence on TCM is important in order to modernise TCM products and expand their clinical usage worldwide.

Xiaoyao decoction (XYD), a conventional herbal formula, has been proven effective and safe for many diseases, such as depressive disorder [8, 9], stress-induced anxiety [7], chronic hepatitis B [8], breast cancer [9], hypertension [10], insomnia [11–14], anovulatory infertility [15] and polycystic ovary syndrome [16]. XYD has also been reported to soothe liver diseases, to invigorate the spleen [17] and to change the content of neurotransmitters, such as serotonin, norepinephrine and substance P [18]. Since clinical applications of XYD have not yet been elucidated clearly at the molecular level, and there are some difficulties in solving the complex system of this herbal formula using experimental methods, the main therapeutic targets and mechanisms of action of this decoction still need to be explored [15].

With the rapid development of bioinformatics, a promising methodology called network pharmacology (NP) has emerged and has been applied to the research of TCM [2]. NP can be used to explore network dynamics and interactions, which coincide with the characteristics of TCM and a holistic view of herbal formulas. In NP-based TCM research, compound–target networks and protein–protein interaction networks are two main types of network analysis. In order to reflect the pathway-based biological effect and to meet the integrated feature of herbal formulas more effectively, this research employed the approach of a targets–pathways–targets (TPT) network and proposed a series of novel network parameters to quantify the integrated effects of herbal formulas on different targets.

**Fig. 1:**
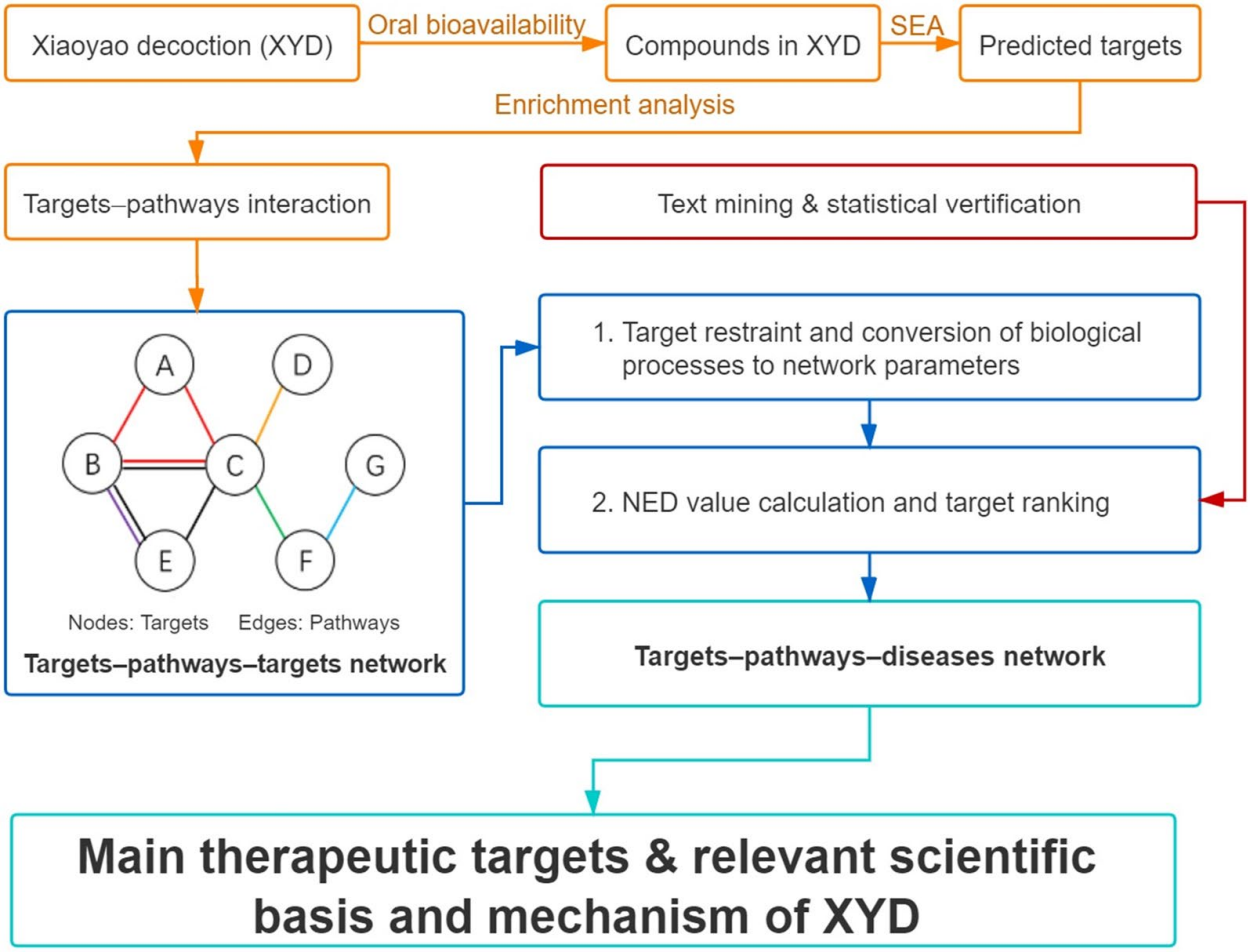
Technology roadmap

In this study we screened the oral bioavailability attributes of chemical data of XYD and obtained the predicted targets; these were the basis of an enrichment analysis to get potential targets and related pathways. The TPT network was established based on the targets–pathways interactions [19]. In the TPT network, the importance of each target was calculated by the declining value of network efficiency, which represents the influential strength of a specific set-off target on the whole network. Furthermore, a “targets–pathways–diseases” network was conducted to identify the scientific basis of XYD. The workflow of the NP-based approach and its application in XYD is shown in Fig. 1.

By taking XYD as an example of classic famous herbal formulas, this study employs the NP-based method to identify the main therapeutic targets of XYD and to provide a methodological reference of clarifying the scientific basis of herbal formulas. This kind of NP-based research is of great significance for translating traditional herbal formulas into modern drugs, given that regulation of TCM products is becoming much stricter nowadays [20, 21].

## Methods

### Data collection and processing

XYD is composed of eight herbs: Radix Bupleuri (*Bupleurum Chinense DC.*), Radix Angelicae Sinensis (*Angelica sinensis (Oliv.) Diels*), Radix Paeoniae Alba (*Paeonia lactiflora Pall.*), Rhizoma Atractylodis Macrocephalae (*Atractylodes macrocephala Koidz.*), Poria (*Poria cocos (Schw.*) *Wolf*), Radix Glycyrrhizae (*Glycyrrhiza uralensis Fisch.*), Herba Menthae Haplocalycis (*Mentha haplocalyx Briq.*) and Rhizoma Zingiberis Recens *(Zingiber officinale Rosc.*). Chemical data of the eight herbs were collected from three chemical databases: the Traditional Chinese Medicine Systems Pharmacology (TCMSP [22]; http://lsp.nwu.edu.cn/tcmsp.php; accessed in August 2019) database; the Compound Reference Database (CRD; http://www.chemcpd.csdb.cn/cmpref/default.html; accessed in August 2019) from the Chinese Academy of Sciences; and the Traditional Chinese Medicines Integrated Database (TCMID [23], http://www.megabionet.org/tcmid/, accessed in August 2019). The common amino acids and compounds with high molecular weight were filtered out from this study. The PubChem database [24] (https://pubchem.ncbi.nlm.nih.gov/; accessed in August 2019) was used to standardise the names and obtain simplified molecular-input line-entry system (SMILES) information for all chemical data.

An increasing number of studies on TCM have found that compounds with favourable therapeutic effects in vitro may not perform well in vivo due to their low oral bioavailability (OB) [25]. In this research, Veber’s filter was used for the OB prediction [26], which meant that the number of rotatable bonds of a given compound had to be ≤ 10; at the same time, the topological polar surface area had to be ≤ 140 Å^2^ [27]. Veber’s filter was also widely used in absorption, distribution, metabolism, excretion (ADME) prediction models [28–30]. SwissADME [31] was used to calculate the molecular properties of the compounds by importing the SMILES information.

The protein targets of compounds from XYD were predicted using a similarity ensemble approach (SEA [32]; http://sea.bkslab.org/), which is a chemical similarity searching-based prediction tool.

The predicted targets were enriched from the Kyoto Encyclopaedia of Genes and Genomes (KEGG) pathways using STRING [33] (http://string-db.org/; version: 11.0) to create the targets–pathways interaction, which laid a foundation for the following network construction and analysis. The enriched pathways with false rate discovery (FDR) < 0.05 were used in the subsequent research.

### TPT network construction

In order to further depict the relationship between the predicted targets, the TPT network was established on the one-mode targets–targets interaction basis that was transferred by Pajek software [34] from the two-mode “targets–pathways” relationship. Nodes of the TPT network were visualised and analysed using Gephi 0.9.2 software [35], which are referred to as protein targets. If two nodes are connected by an edge, this means they were both involved in at least one of the same pathways.

### Key target analysis

We considered the TPT network as an integral network as a whole where XYD took effect. When a target was not disturbed by XYD directly, the relative pathways would be affected. The edges would then be affected by these pathways, finally resulting in a change of the whole network. Hence, each selected target would have a different impact on the entire network.

### Target restraint and network parameters

Firstly, considering the influence of the target on pathways and the convenience of calculation, we assumed the contribution of each target in the pathways to be consistent. Therefore, if a pathway had N_j_ targets, the effect of one set-off target on the pathway was $\frac{1}{{N_{j} }}$, and the efficacy of the pathway became $\frac{{N_{j} - 1}}{{N_{j} }}$ after setting off the target. Conversely, if the set-off target was unrelated to the pathways, the efficacy of the unaffected pathways was 1.

To compute the efficacy of the edge, we needed to know which pathways the edge contained, whether these pathways were affected or not, and what the respective efficacy of the affected pathways was. The efficacy of the edge was equal to the product of the efficacy of all the pathways. Since the unaffected pathway efficacy was 1, the final efficacy of the edge was equal to the product of all affected pathways’ efficacy. For example, if an edge had *p* pathways, where *t* pathways were affected, the edge efficacy (EE) would be equal to $\frac{{N_{1} - 1}}{{N_{1} }} \times \cdots \times \frac{{N_{t} - 1}}{{N_{t} }}$. The length of the affected edges was $\frac{1}{EE}$ [36], and the length of the unaffected edges was 1.

### Network efficiency decrease (NED)

Network efficiency (NE) was calculated for every TPT network after restraining every target in the sequence [37]. NE was defined as the sum of the reciprocals of the shortest path lengths between all pairs of nodes and reflected the integrity of the whole network. The programme of network efficiency calculation was written in Python 3.0 with a modified Dijkstra algorithm made by the authors. The importance of T_i_ can be measured by NED_i_; i.e., NE_0_–NE_i_. All symbols are described in Table 1.

**Table 1:** List of symbols

Target	Pathway efficacy (PE)	Edge efficacy (EE)	Length of edge (L)		Network efficiency (NE)	Network efficiency decrease (NED) value
T_i_	$PE = \frac{{N_{j} - 1}}{{N_{j} }}$	$EE = \frac{{N_{1} - 1}}{{N_{1} }} \times \cdots \times \frac{{N_{t} - 1}}{{N_{t} }}$	$L_{affected} = \frac{1}{EE}$	$L_{unaffected} = 1$	$NE_{i} = \mathop \sum \limits_{m \ne n \in G} \frac{1}{{d_{mn} }}$	$NED_{i} = NE_{0} - NE_{i}$

*T*_*i*_ setting-off target i, *N*_*j*_ the number of targets in a pathway, *t* the number of affected pathways in an edge, *G* a set of nodes in a graph, *m, n* nodes in a graph, $d_{mn}$ the shortest path length between nodes m and n, *NE*_*0*_ original network efficiency, *NE*_*i*_ network efficiency after setting off target i

To illustrate the NED calculation within a network, a sample TPT network was constructed to show the calculation process.

**Fig. 2:**
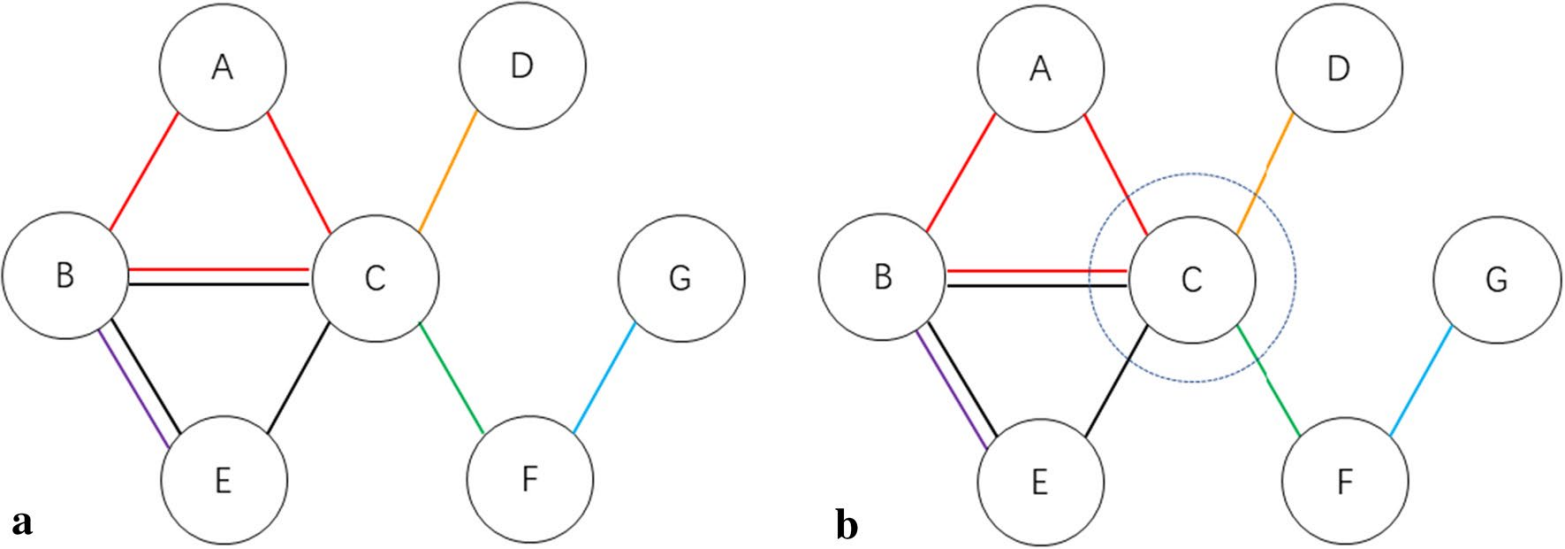
**a** The sample TPT network; **b** the sample TPT network with target C restrained. The nodes represent targets and the edges represent pathways

Figure 2a shows the original network with seven targets and six pathways. We chose target C as our set-off target, shown in Fig. 2b. The relative pathways would also be affected. The extent of the effect depended on the target number in one pathway. Then, the PE of pathway a and pathway b decreased to 67% while the efficacy of pathway d and pathway e decreased to 50%. Details of the affected pathways information are shown in Table 2.

**Table 2:**
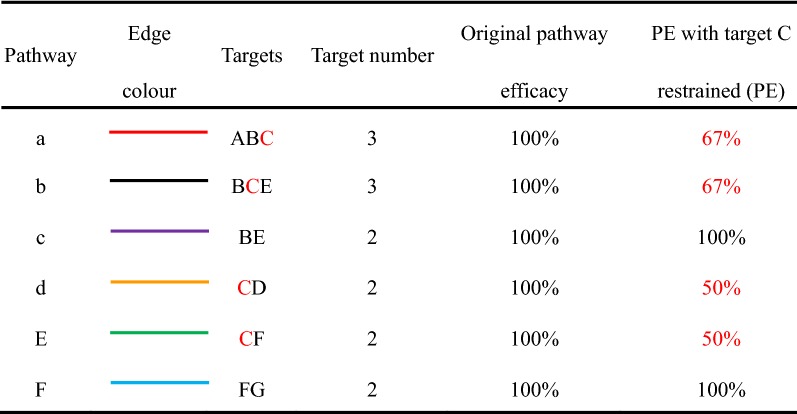
Information in the sample TPT network

In the following step, pathway efficacy was transferred into edge efficacy and the length of the edge. As shown in Table 3, every edge efficacy was calculated by the specific pathways in the edge and their corresponding PE. The length of the edge was equal to the reciprocal of the edge efficacy. Finally, a distance matrix was constructed, as shown in Table 4. Using a Dijkstra algorithm, the NE of the sample TPT network with target C restrained was calculated. The corresponding NED value would be obtained in the last step. In this sample network, NE_0_ = 13.83, NE_target C_ = 8.60 and NED_target C_ = 5.23.

**Table 3:** Edge efficacy and length of edges after setting off target C

Edge	Affected edges							Unaffected edges
	AB	AC	BC	BE	CD	CE	CF	FG
Pathway	a	a	a, b	b, c	d	b	e	f
EE	67%	67%	67%*67%	67%*100%	50%	67%	50%	100%
L	1.5	1.5	2.25	1.5	2	1.5	2	1

**Table 4:** Matrix of length of edge

	A	B	C	D	E	F	G
A	M	1.5	1.5	M	M	M	M
B	1.5	M	2.25	M	1.5	M	M
C	1.5	2.25	M	2	1.5	2	M
D	M	M	2	M	M	M	M
E	M	1.5	1.5	M	M	M	M
F	M	M	2	M	M	M	1
G	M	M	M	M	M	1	M

M means two nodes are not connected directly in the Dijkstra algorithm

### Targets–pathways–diseases (TPD) network construction

According to the TPT network and the corresponding analysis, we found relatively important targets from all predicted targets, and further analysed the pathways they focused on and the relative diseases. As a credible database, the Kyoto Encyclopaedia of Genes and Genomes [38] (KEGG; http://www.genome.jp/kegg/pathway.html) was employed to identify the human diseases using International Classification of Diseases, 11th Revision (ICD-11) classification. Herein, we built a targets–pathways–diseases directed network and got more significant pathways and diseases according to the network parameters. The network was produced using Cytoscape 3.7.0 software [39]. The nodes represented the main targets, pathways and diseases. The edges indicated the relationship between two different category nodes.

### Text mining and statistical verification

After retrieving data from the TPT network via key target analysis, a network-based approach was employed to elucidate the main targets of a formula after passing a statistical test. Based on the co-occurrence matrix, text mining has a wide application for different purposes, such as integrating disease-gene associations [40] and extracting the known biology [41]. To collect the data of associations between XYD and the relevant targets in the existing literature, the rationality of the predicted target was further validated by testing the correlation between topological scores and co-occurrence intensity. Due to the particularity of the Chinese formula, research about XYD in the present study was mainly from the Chinese literature database, China National Knowledge Infrastructure (CNKI; https://www.cnki.net/; accessed in September 2019). We retrieved the number of relative literatures from CNKI by searching “FT = ‘逍遥散’*‘gene name’”(FT, full text; 逍遥散, the Chinese name of XYD); for example, “FT = ‘逍遥散’*‘AKT1’”. For abstracts, we searched “AB = ‘逍遥散’ *‘gene name’”. In graph theory, centrality could be applied to characterise the importance of the nodes in a network. Hence, we chose two centralities, degree centrality (DC) and betweenness centrality (BC), as two vital network parameters of our evidence [42–44]. Using SPSS 24.0, we performed Spearman’s test on NED, DC and BC for both abstract and full-text searching.

## Results

### TPT network

XYD is made of eight herbs and a total of 1542 compounds. After data processing, 839 sample chemicals exhibited better oral bioavailability. 347 targets were predicted for further analysis using the SEA method.

**Fig. 3:**
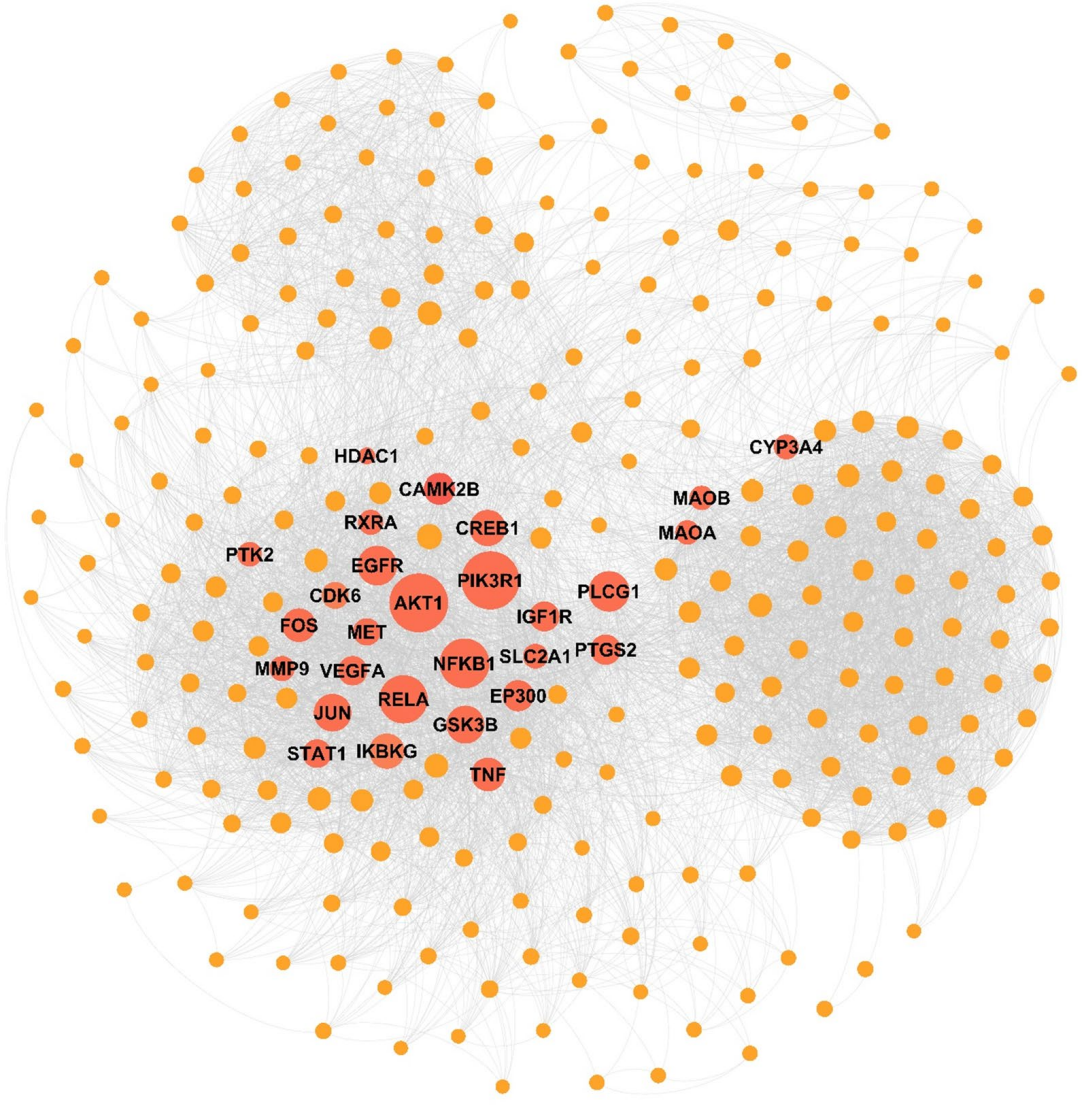
TPT network. Yellow nodes stand for subordinate targets; orange nodes stand for main targets; the size of the nodes stands for their NED value; grey lines stand for the associations between nodes based on pathways; i.e., two nodes are linked if they have at least one common pathway

After enrichment analysis using STRING, a total of 155 highly related pathways were identified with 279 relevant potential targets. Based on these data, a TPT network of targets–pathways interaction in XYD treatment was constructed, as shown in Fig. 3. Please refer to the supplementary dataset for more detailed information about the compounds (Additional file 1: Chemicals data), putative targets (Additional file 1: Putative targets) and relevant pathways (Additional file 1: Targets–pathways relationship).

Based on the TPT network and key targets analysis, we ranked the targets according to the NED value. We chose the top 10% of targets as the main targets that might have primary effects. Detailed information of the top 10% of targets is shown in Table 5. Please refer to Additional file 1: Final results for detailed information about all NED results.

**Table 5:** Top 10% of NED targets

Gene	NE	NED	Gene	NE	NED
AKT1	20,333.65	594.72	PTGS2	20,708.19	220.17
PIK3R1	20,346.31	582.06	IGF1R	20,712.46	215.90
NFKB1	20,451.82	476.54	VEGFA	20,721.39	206.97
RELA	20,471.79	456.58	STAT1	20,730.23	198.14
PLCG1	20,573.21	355.16	MET	20,747.77	180.60
EGFR	20,588.51	339.85	CDK6	20,753.56	174.80
GSK3B	20,608.93	319.43	MMP9	20,776.22	152.15
JUN	20,612.22	316.14	RXRA	20,776.68	151.69
CREB1	20,630.58	297.78	HDAC1	20,777.35	151.02
IKBKG	20,642.31	286.06	CYP3A4	20,778.75	149.62
TNF	20,664.10	264.26	SLC2A1	20,780.26	148.11
FOS	20,665.18	263.19	PTK2	20,783.19	145.17
EP300	20,696.71	231.66	MAOA	20,785.74	142.63
CAMK2B	20,696.78	231.59	MAOB	20,785.74	142.63

NE_0_ = 20,928.37

### TPD network

**Fig. 4:**
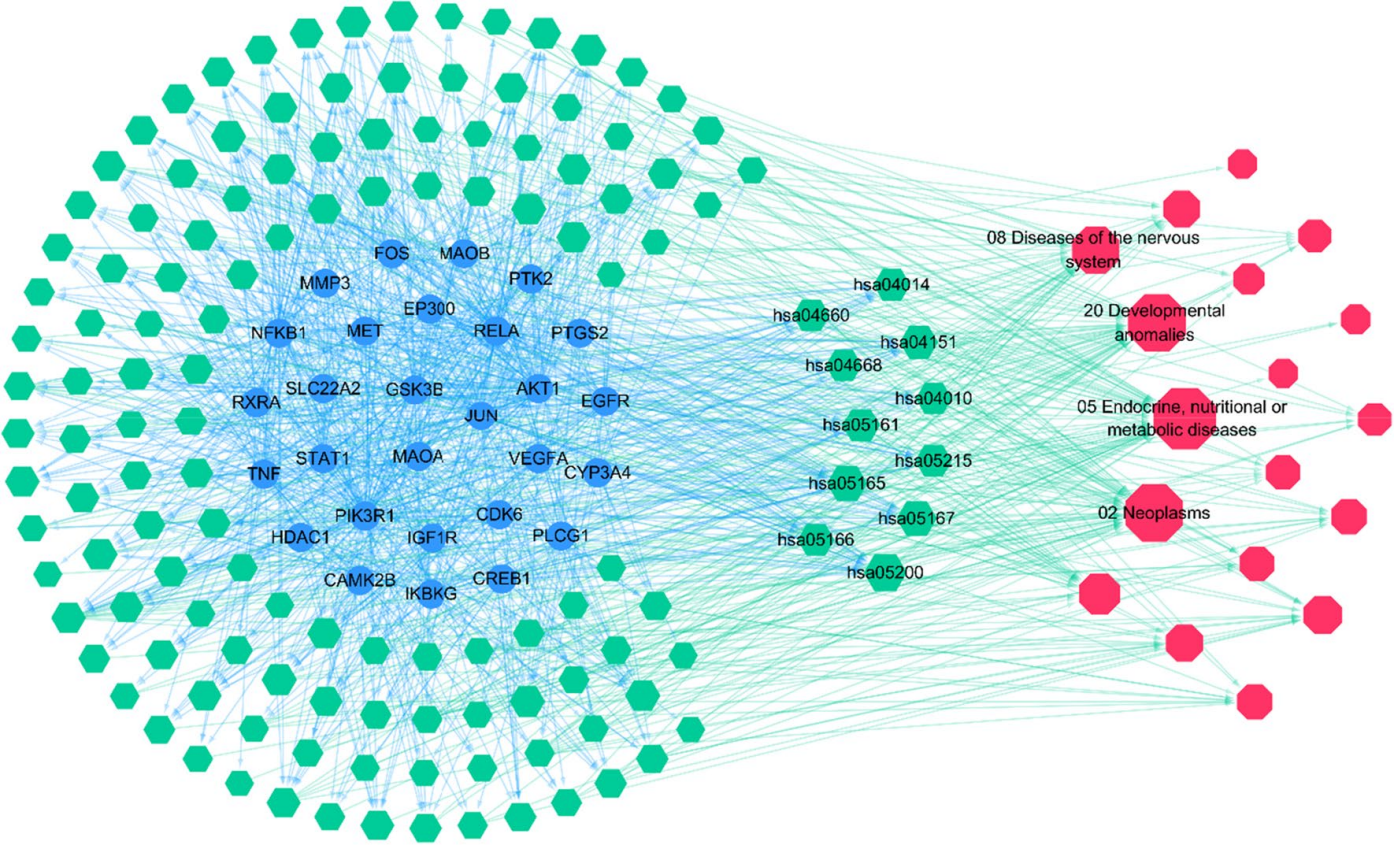
TPD network. Blue circles represent main targets; green hexagons represent correlative pathways and the most important cluster (DC > 10) is labelled; red octagons represent relevant diseases with four more important nodes labelled; the size of the nodes represents their degree centrality in the network; blue lines represent the correlation of targets and pathways, and green lines represent the links of pathways and diseases

According to the target–pathway interactions, the TPD network was constructed. We found main targets in 134 pathways out of a total of 155 pathways. Eleven main pathways with an indegree of > 10 were identified. Detailed information of the main pathways is shown in Table 6. According to the indicator of the network, 05 endocrine, nutritional or metabolic diseases, 20 developmental anomalies, 02 neoplasms and 08 diseases of the nervous system with an indegree of > 20. In Fig. 4, blue circles represent the main targets, green hexagons represent the correlative pathways and red octagons represent the relevant diseases. The size of the nodes represents their degree centrality in the network. The most important pathways (DC > 10) and four primary diseases are labelled. Blue lines represent the correlation between targets and pathways and green lines represent the links of pathways and diseases. Please refer to Additional file 1: Pathways–diseases relationship for detailed information about the pathways–diseases relationship.

**Table 6:** Pathways with high indegree value

Pathway ID	Pathway name	FDR	Indegree
hsa05200	Kaposi’s sarcoma-associated herpesvirus infection	2.23 × 10^−16^	21
hsa05167	Human papillomavirus infection	1.87 × 10^−7^	17
hsa05165	Hepatitis B	1.16 × 10^−6^	17
hsa05161	PI3K–Akt signalling pathway	3.96 × 10^−6^	14
hsa04151	Prostate cancer	3.96 × 10^−6^	14
hsa05215	HTLV-I infection	2.07 × 10^−8^	12
hsa05166	T cell receptor signalling pathway	2.15 × 10^−5^	12
hsa04660	Ras signalling pathway	2.17 × 10^−8^	12
hsa04014	TNF signalling pathway	2.60 × 10^−6^	12
hsa04668	MAPK signalling pathway	1.46 × 10^−6^	11
hsa04010	Fluid shear stress and atherosclerosis	3.96 × 10^−6^	11

FDR means false discovery rate

Blue circles represent main targets; green hexagons represent correlative pathways and the most important cluster (DC > 10) is labelled; red octagons represent relevant diseases with four more important nodes labelled; the size of the nodes represents their degree centrality in the network; blue lines represent the correlation of targets and pathways, and green lines represent the links of pathways and diseases.

### Statistical verification

Spearman’s test showed significant results (two-tailed) in both full texts and abstracts with NED, DC and BC (*p *< 0.001). As shown in Table 7, NED had a better performance in this statistical test than DC and BC, with a smaller *p* value and higher correlation coefficient. Please refer to Additional file 1: Co-occurrence verification for detailed information about the NED, DC and BC of all targets.

**Table 7:** Results of Spearman’s correlation test

		NED	DC	BC
Full text	Correlation coefficient	0.388**	0.346**	0.316**
	*p*-value	0.000	0.000	0.000
Abstract	Correlation coefficient	0.230**	0.202*	0.182*
	*p*-value	0.000	0.001	0.002

** Means that the correlation coefficient is significant at the 0.001 level (two-tailed), while * shows significance at the 0.01 level (two-tailed)

## Discussion

TCM formulas always contain multiple herbs (multiple components) and exert a holistic influence through the “multi-component, multi-target, multi-effect” mechanism [3]. It is the complexity of TCM that has led to the difficulty of studying it. Lack of information about active pharmacologic principles [45], the suboptimal quality of some herbal products [46] and a lack of clinical evidence [47] make TCM unable to meet the regulatory requirements set for modern drugs, which are now widely accepted throughout the world. However, despite being questioned, TCM, with 5000 years of inheritance, has never been eliminated since its inception and, presently, 1.5 billion people still use TCM as healthcare supplements or preventive medicine [48], suggesting that it still has strong vitality and significant demand. Hence, how to fulfil the regulatory requirements that are set for modern medicine is the most critical problem that TCM is currently facing. One of the core actions to tackle the challenge is to investigate the pharmacological mechanisms of TCM.

In molecular biology, one target could affect multiple pathways, and each pathway could be affected by multiple targets. By combining biological information with network analysis, we proposed a novel network parameter to evaluate the primary nodes in the TPT network. We considered these targets as main targets, which play dominant roles in this network.

In this study, we assumed that a set-off target could not be disturbed by XYD. After restraining the set-off target, we obtained a new TPT weighted network. Compared with the original TPT network, the effectiveness of the pathways associated with the target was reduced from a biological point of view. In the TPT network, all edges were composed of different pathways, and the decrease of pathway efficacy led to the decrease of edge efficacy. This change also led to the increase of edge length, and the network became sparser than the original network, eventually leading to the decrease of network efficiency. Finally, we got a network efficiency decline (NED) value from every new TPT network and compared this value to evaluate the influence of each target on the original network. From a statistical perspective, Spearman’s test of NED showed better performance than DC and BC did, indicating that NED is a better indicator.

Generally, the association of the top ranking (main targets) group and relevant pharmacological studies of XYD have been extensively discussed in the existing literature. For instance, AKT1 (threonine-protein kinase), which plays a role in cell growth, survival, metabolism, carcinogenic transformation and other biological processes, takes part in the alleviative effect of XYD on the autophagy of granulosa cells induced by chronic unpredictable mild stress (CUMS) in vivo [48], affecting follicle development [16, 49]. It is also associated with the amelioration of stress-induced abnormal levels of insulin, blood glucose, cholesterol (CHOL), low density lipoprotein cholesterol (LDL-C) and high density lipoprotein cholesterol (HDL-C) [50]. Cyclic adenosine monophosphate-responsive element-binding protein 1 (CREB1), as a representative indicator of the hippocampus of depressed rats, was reported to implicate depression via down-regulation, and thus was considered as the potential target of XYD treatment [51]. Tumour necrosis factor (TNF) is related to a TNF-α pathway, which was found to be down-regulated by XYD to exert anxiolytic-like effects on chronic immobilization stress-induced anxiety [52]. FOS (proto-oncogene c-Fos), which is closely related to signal transduction, has shown to be an indicator for the potential of XYD to regulate the activity of the sympathetic nervous system, attributed to the increased expression of FOS during XYD treatment [16].

In the end, by analysing the topological properties of the TPD network, we found that XYD could play a therapeutic role in many complicated diseases, such as in 05 endocrine, nutritional or metabolic diseases, 20 developmental anomalies, 08 diseases of the nervous system and two neoplasms. For instance, there are several important pathways included in the five endocrine, nutritional or metabolic diseases, such as the fluid shear stress [53] and atherosclerosis pathway [54], the prostate cancer pathway [55], the Ras signalling pathway [56] and the mitogen-activated protein kinase (MAPK) signalling pathway [57], etc. The relationships between pathways and diseases demonstrated multiple pathways in eight diseases of the nervous system, including the fluid shear stress [58] and atherosclerosis pathway [59], the prostate cancer pathway [60], the Ras signalling pathway [61] and the T cell receptor signalling [62] pathway, etc. Also, eight of the eleven important pathways are mentioned in two neoplasms. Currently, there are not sufficient studies on XYD related to developmental anomalies, which could be a novel therapeutic area based on our research.

For the top-ranking group, we further identified active compounds related to the main targets based on the SEA results. Several specific compounds show effects on multiple targets in this group. For example, Quercetin (Pubchem CID: 5280343), derived from Radix Glycyrrhizae (*Glycyrrhiza uralensis Fisch.*) and Radix Bupleuri (*Bupleurum Chinese DC.*), can take effect on AKT1, PIK3R1, EGFR, GSK3B, CAMK2B, IGF1R, MET, MMP9, PTK2 and MAOA. Ferulic acid (Pubchem CID: 709), derived from Radix Angelicae Sinensis (*Angelica sinensis (Oliv.) Diels*) and Herba Menthae Haplocalycis (*Mentha haplocalyx Briq.*), is effective for NFKB1, FOS, EP300, MMP9, MAOA, JUN, MAOB, IKBKG and MET. Similarly, apigenin (Pubchem CID: 5280443) from Herba Menthae Haplocalycis (*Mentha haplocalyx Briq.*), Coniferyl ferulate (Pubchem CID: 6441913) from Radix Angelicae Sinensis (*Angelica sinensis (Oliv.) Diels*) and 6-dehydrogingerdione (Pubchem CID: 5316449) and 1, 2-dihydrocurcumin (Pubchem CID: 5372374) from Rhizoma Zingiberis Recens (*Zingiber officinale Rosc.*) are all related to more than five main targets. It would be significant work to investigate the effects of these compounds on related targets by further pharmacological experiments in terms of the importance of these compounds in the decoction.

Meanwhile, 11 pathways were considered to be important in our study. Some of the pathways have been shown to be associated with pharmacologic mechanisms of various diseases. Generally, XYD can treat liver diseases through the galactose metabolism pathway [63], the hepatitis B pathway [64], the PI3K–Akt signalling pathway [65] and the MAPK signalling pathway [66–69]. Pathway enrichment analyses also demonstrated XYD to be involved in the regulation of multiple pathways of inflammatory, including the kaposi sarcoma-associated herpesvirus infection pathway [70], the T cell receptor signalling pathway [71] and the tumour necrosis factor pathway. Besides, our study verified that XYD has pharmacologic activities against cancer through the kaposi sarcoma-associated herpesvirus infection pathway [72], the human papillomavirus infection pathway [73], the PI3K–Akt signalling pathway [65] and the Ras signalling pathway [74]. Due to the complexity of TCM formulas, these herbs with different targets and pathways can act on various aspects of the disease through systems.

Several limitations of this study should be noted. Firstly, the SEA method, based on the two-dimensional (2D) structures (SMILE information) of compounds, might generate the same potential targets of the isomeric compounds. Therefore, it is not comprehensive enough to predict solely using the SEA method or any other chemical similarity searching-based prediction method when there are some isomeric compounds in the study. Secondly, further work should conduct an experimental verification to validate the conclusions drawn in this study.

## Conclusions

By combining the biological processes displayed in the network and the topology parameters of the TPT network itself, it is assumed that a single target can cause a decrease in network efficiency of the overall network to determine the contribution of the assumed target to the network. With this approach, further statistical verification demonstrated that the results obtained from the NED values were consistent with the research tendency of the XYD therapeutic targets, and the performance was better than those obtained from DC and BC methods that have been widely used. We selected the top 10% of targets as the main targets and found that they act on 134 of the 155 pathways. As a result, we inferred that the main targets were highly associated with the underlying mechanism of XYD. Some of these targets have been validated as targets for XYD by evidence-based experimental studies. Herein, the network-based method provided a facile and reliable strategy for uncovering the potential therapeutic targets of XYD.

TCM generally lacks evidence-based medical studies; therefore, the mechanism of actions and material connotations are mostly unclear. These issues are significant barriers on the journey of transformation into modern drugs. As a new analytical method, network pharmacology has been widely employed in the investigations of the mechanism of actions and material basis of TCM. The present study has identified the core group of targets among many potential ones based on the holistic and systematic actions of TCM, which can bring insight to the efforts of modernising TCM and the methodology of studying various TCM formulas.

## Supplementary information

**Additional file 1.** Chemicals data.

## Data Availability

The datasets used and/or analysed during the current study are available from the corresponding author on reasonable request.

## Abbreviations

TCM: Traditional Chinese medicine
XYD: Xiaoyao Decotion
TPT: Targets–pathways–targets
NP: Network pharmacology
TCMSP: Traditional Chinese medicine systems pharmacology
TCMID: Traditional Chinese medicines integrated database
OB: Oral bioavailability
SEA: Similarity ensemble approach
FDR: False rate discovery
NE: Network efficiency
EE: Edge efficacy
PE: Pathway efficacy
TPD: Targets–Pathways–Diseases
NED: Network efficiency decline
